# The curative effect analysis of a modified Kirschner wires and locking plate internal fixation method for the fifth metacarpal neck fracture

**DOI:** 10.1186/s13018-021-02627-8

**Published:** 2021-08-12

**Authors:** Song Gu, Long Zhou, Yinjun Huang, Renguo Xie

**Affiliations:** 1 Trauma Center, Shanghai General Hospital, Shanghai Jiao Tong University School of Medicine, NO.650 Xin Songjiang Road, Shanghai, 201620 People’s Republic of China; 2 Department of Obstetrics and Gynecology, Shanghai General Hospital, Shanghai Jiao Tong University School of Medicine, NO.650 Xin Songjiang Road, Shanghai, 201620 People’s Republic of China

**Keywords:** Hand injury, Metacarpal neck fracture, Internal fixation, Curative effect, Kirschner wire

## Abstract

**Purpose:**

To evaluate the efficacy of a modified internal fixation method for the treatment of fifth metacarpal neck fracture.

**Methods:**

From March 2018 to December 2019, 12 patients with the fifth metacarpal neck fractures of the hands were treated with the Kirschner wires and locking plate internal fixation method. Each patient’s gender, age, dominant hand, injured hand, trauma mechanism, preoperative and postoperative deformity (angulation and the length of the fifth metacarpal), the range of motion of the metacarpophalangeal joint and grip strength of each side, the time of return to work, and follow-up time were recorded and calculated.

**Results:**

The mean follow-up time was 16.8 months, and the angulations of preoperative and postoperative deformity were 40.0 ± 3.7°and 17.6 ± 1.7°, respectively. The length of the fifth metacarpals of preoperative and postoperative deformity were 51.5 ± 2.1 mm and 60.0 ± 1.8 mm, respectively. At the last follow-up, the range of motion of the fifth metacarpophalangeal joint of the injured side and the contralateral side were 84.3 ± 3.6°and 86.5 ± 2.0°, and the grip strength of the injured side and the contralateral side were 74.8 ± 6.1 LB and 78.6 ± 8.3 LB, respectively, without statistically significant differences. QDASH score was 2.0 ± 1.0, and the time of return to work was 6.0 ± 0.7 weeks.

**Conclusion:**

The modified internal fixation method is one of the alternative treatments for the fifth metacarpal neck fracture with good curative effects.

## Introduction

The fracture of the fifth metacarpal neck is very common in the clinical work of orthopedics and hand surgery [1–3]. The controversy over the treatment of the fifth metacarpal neck fracture has never ceased. Some scholars hold the view that angulation (more than 30) can lead to a decrease of flexion force in extrinsic tendons and loss of grip strength [4, 5], and this kind of fracture should be treated surgically. Kirschner wire fixation and plate fixation are two common surgical treatment methods [6–9]. For the old fifth metacarpal neck fractures with the failure of closed reduction, open reduction and plate internal fixation are often required. The use of the medial locking plate was reported [10] in the treatment of the fifth metacarpal neck fracture, and two screws were inserted into the distal part of the fracture, which could achieve satisfactory effects.

However, the fracture line of the fifth metacarpal neck is always very close to the metacarpal head, and there is often not enough space to place two screws in the distal part of the fracture. However, one screw is not stable enough to guarantee the fixation strength. To solve this problem, we adopted a new method of fixation with Kirschner wires and locking plates. The aim of this study was to retrospectively analyze the results of the modified internal fixation method for the treatment of fifth metacarpal neck fracture.

## Materials and methods

From March 2018 to January 2019, 12 patients suffering from the fifth metacarpal neck fractures of the hands were treated by adopting the modified internal fixation method. The inclusion criteria included that (1) closed fifth metacarpal neck fracture, (2) closed reduction of the fracture failed, (3) the angulation of the longitudinal axis of the proximal and distal sides of the fracture was more than 30°, and (4) there was no other injury in the hand. The exclusion criteria included that (1) open fifth metacarpal neck fracture, (2) closed reduction of the fracture was successful, (3) the angulation of the longitudinal axis of the proximal and distal sides of the fracture was 30° and below, and (4) there were other injuries in the hand. Table 1 lists the demographic data of the 12 patients.

**Table 1:** Demographic data of patients

Case	Sex	Age	Dominant hand	Injuried hand	Trauma mechanism	Preoperative deformity		Postoperative deformity	
						angulation (°)	length (mm)	angulation (°)	length (mm)
1	M	62	R	R	Sports injury	41	52	15	60
2	M	56	R	R	Traffic injury	40	55	18	61
3	M	34	L	L	Traffic injury	42	52	17	59
4	M	38	L	R	Sports injury	45	49	20	57
5	M	27	R	R	Sports injury	38	51	15	62
6	M	32	R	R	Sports injury	35	55	17	62
7	F	32	L	R	Sports injury	40	52	18	61
8	M	17	R	R	Sports injury	39	49	18	58
9	M	33	R	R	Traffic injury	38	53	19	61
1o	M	40	R	R	Traffic injury	35	50	18	59
11	M	45	L	R	Sports injury	39	51	16	62
12	M	39	R	R	Sports injury	48	49	20	58

Length: the length of the fifth metacarpal

### Surgical technique

**Fig. 1:**
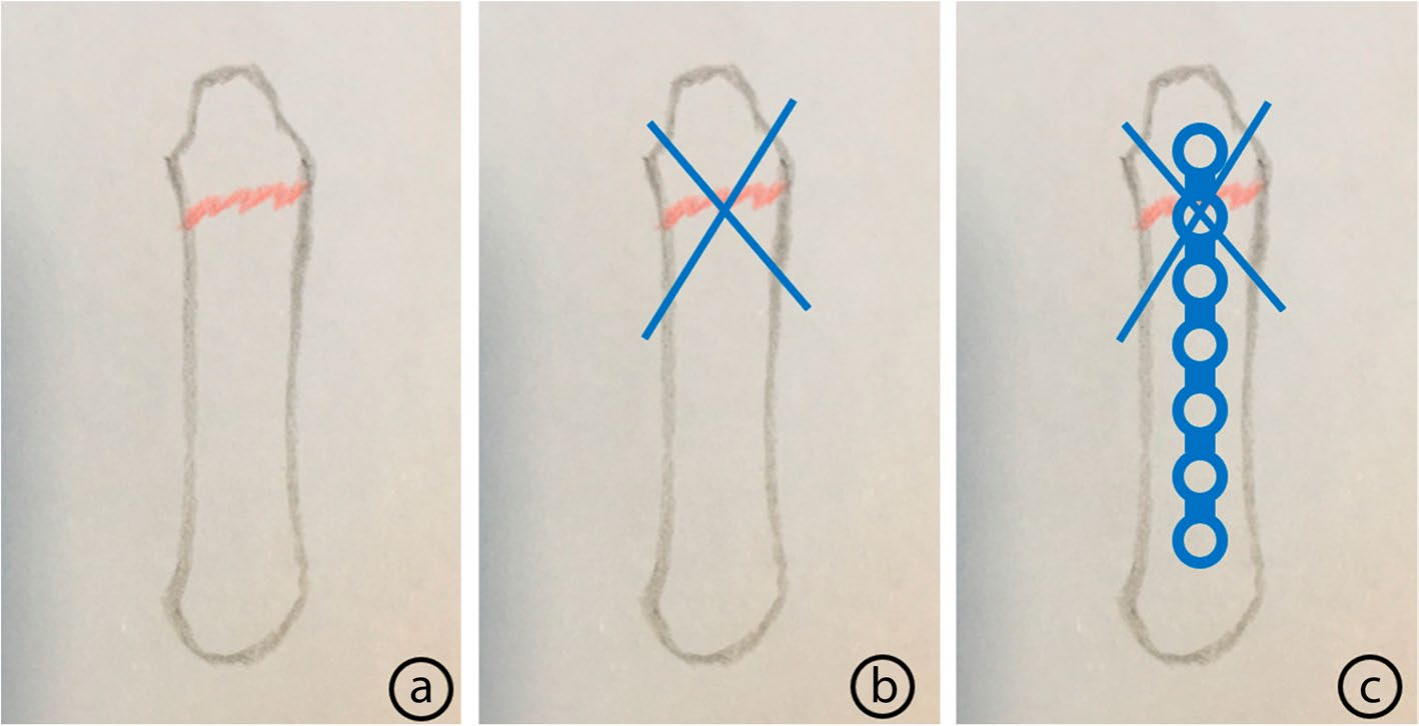
The schematic diagram of surgical procedure. **a** The fracture reduction was achieved successfully. **b** Two Kirschner wires were used to fix the fracture. **c** The plate was inserted with three to four screws in the proximal part of the fracture and one screw in the distal part

Each operation was performed with local anesthesia and an electric tourniquet. A dose of 3 ml lidocaine was injected every 1.5–2 cm from the base of the metacarpal bone to the distal end and the injection needle was inserted vertically to the surface of the metacarpal bone. A dorsal incision was made, and the fracture was exposed through the space between the extensor tendons. The scars and hyperplasia around the fracture were removed thoroughly. One surgeon pulled the little finger to the distal side to reduce the fracture and flexed the metacarpophalangeal joint (Fig. 1a), while another surgeon inserted cross Kirschner wires to fix the fracture (Fig. 1b). Both Kirschner wires were inserted from the distal part to the proximal part of the fracture and penetrated two layers of cortical bone. The first Kirschner wire was inserted from the ulnar side to the radial side and the second was opposite. After the Kirschner wire was inserted, the excess part of the tail needed to be cut off. Generally, 3–5 mm was left on the outside of the metacarpal head, so as to facilitate the removal of internal fixation and minimize the impacts on the metacarpophalangeal joint capsule and extensor retinaculum. Due to local anesthesia, the patients could actively move the joint during the operations, and the unaffected extension and flexion functions were the main references for the length of the Kirschner wire tail. After the reduction was confirmed to be satisfactory by C-arm fluoroscopy, a straight F3 locking plate (Zimmer Biomet, Warsaw, Indiana, USA) was inserted. Three to four screws were placed in the proximal part of the fracture and one screw was placed in the distal part (Fig. 1c). If necessary, part of the joint capsule could be appropriately opened to make sure that the plate and screw could be placed. Afterwards, the joint capsule should be sutured with 4-0 Coated Vicryl Plus Antibacterial Suture (Ethicon, Somerville, NJ, USA). Each patient was asked to move his or her fingers intraoperatively, so as to ensure that the range of extension and flexion motion was not limited.

### Postoperative management

Two weeks after the operation, the suture was removed and the function exercise started. All patients underwent function exercise under the guidance of the same group of physiotherapists. The extension and flexion activities of the interphalangeal joint of the little finger were mainly conducted 2 to 3 weeks after the surgeries. The extension and flexion activities of the metacarpophalangeal and wrist joint began 3 weeks after the surgeries. X-rays were examined every 3–4 weeks after the operation. About 3–4 months after the operation, the fracture got healed and the internal fixation was removed. The function exercises were continued.

### Outcome evaluation

In this study, each patient’s gender, age, dominant hand, injured hand, trauma mechanism, the duration of the surgery, preoperative and postoperative deformity (angulation and the length of the fifth metacarpal), the range of motion of the metacarpophalangeal joint and grip strength of each side, the time of return to work, and follow-up time were recorded. Postoperative complications such as the failure of internal fixation, fracture nonunion, and fracture malunion were recorded. Grip strength was measured using a Jamar dynamometer (Baseline Hydraulic Hand Dynamometers; Fabrication Enterprises, White Plains, NY, USA). According to the QDASH (the Quick Disability of the Arm, Shoulder and Hand) score, we evaluated the postoperative function. Another group of surgeons who did not participate in the surgeries performed these assessments. The mean and standard deviation of the preoperative and postoperative deformity and the time of return to work and follow-up were calculated. Meanwhile, the range of motion of the metacarpophalangeal joint and grip strength of both sides were calculated, and one-way analysis of variance was carried out. *P* value of < 0.05 was defined to be statistical significant, and SPSS 21.0 software was applied for statistical analysis.

## Results

**Fig. 2:**
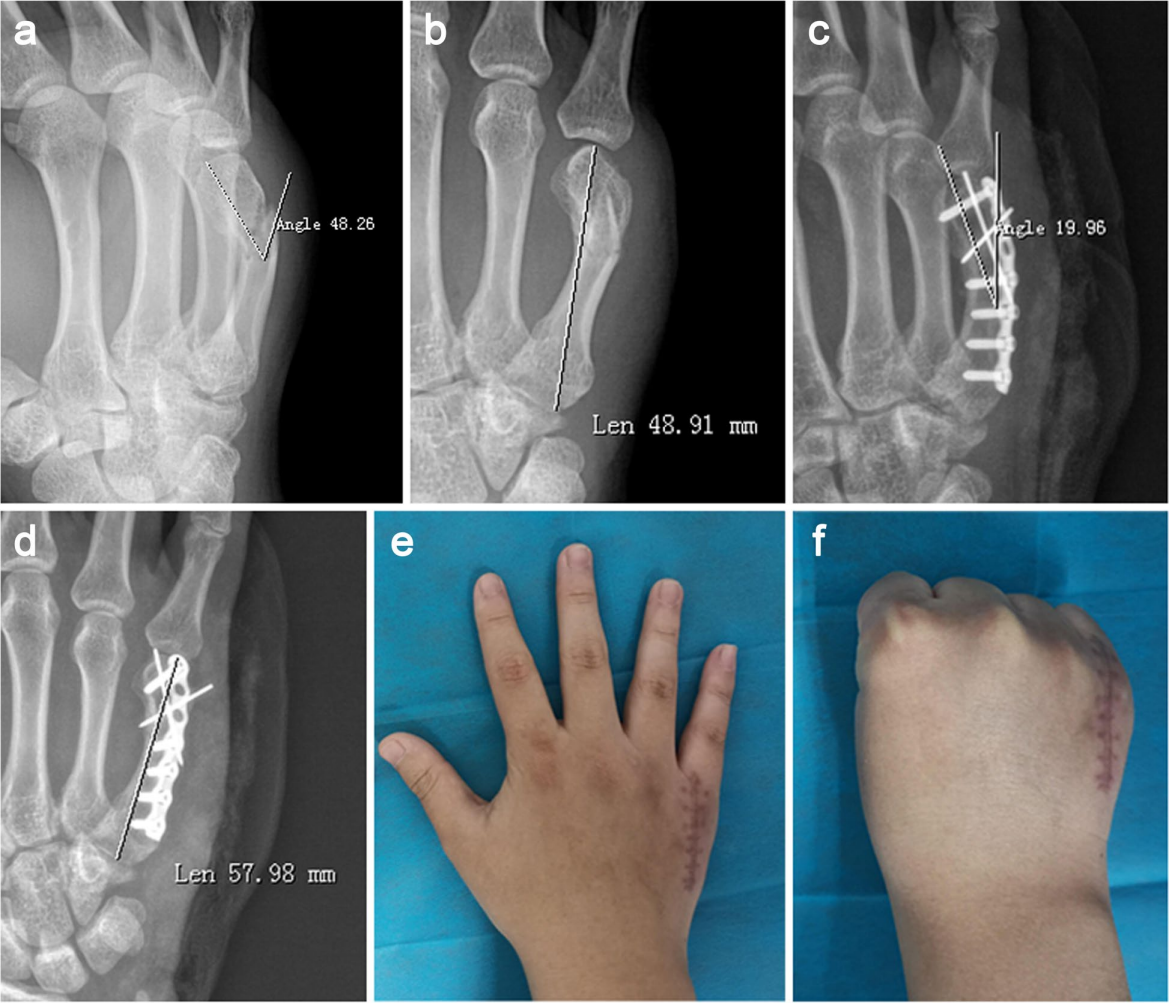
The treatment process of patient No.12. **a** The preoperative lateral X-ray with the angulation of 48°. **b** The preoperative anteroposterior X-ray with a length of the fifth metacarpal of 49 mm. **c** The postoperative lateral X-ray with the angulation of 20°. **d** The postoperative anteroposterior X-ray with a length of the fifth metacarpal of 58 mm. **e** The appearance of the extension position of the fifth metacarpophalangeal joint at the last follow-up. **f** The appearance of the flexion position of the fifth metacarpophalangeal joint at the last follow-up

The mean follow-up time was 16.8 months (ranging from 14 to 18 months) and the mean surgery duration was 44.8 min (ranging from 35 to 55 min) (Table 2). The angulations of preoperative (Fig. 2a) and postoperative (Fig. 2b) deformities were 40.0 ± 3.7° and 17.6 ± 1.7°, respectively. The length of the fifth metacarpals of preoperative (Fig. 2c) and postoperative (Fig. 2d) deformity were 51.5 ± 2.1 mm and 60.0 ± 1.8 mm, respectively. At the last follow-up, the range of motion of the fifth metacarpophalangeal joint of the injured side (Fig. 2e, f) and the contralateral side were 84.3 ± 3.6° and 86.5 ± 2.0°, respectively, and the differences were not statistically significant. The grip strength of the injured side and the contralateral side were 74.8 ± 6.1 LB and 78.6 ± 8.3 LB, respectively, and the differences were not statistically significant. QDASH score was 2.0 ± 1.0, and the time of return to work was 6.0 ± 0.7 weeks (Table 3). In the 12 cases, each case had some degree of adhesion between the extensor tendon and the locking plate, and arthrolysis and tendonolysis were conducted when the internal fixation was removed. No incision infection occurred, and each incision got healed.

**Table 2:** Clinical and functional outcomes

Case	Follow up time (months)	surgery duration (min)	Range of motion (°)		Grip strength (LB)		QDASH score	Return to work(weeks)
			TIS	TCS	TIS	TCS		
1	14	45	85	90	80	85	3	5
2	14	40	83	86	83	86	2	6
3	16	45	87	88	76	90	1	6
4	16	50	90	90	77	88	2	6
5	17	45	81	85	82	84	3	6
6	17	50	79	85	72	70	4	5
7	17	55	80	85	69	75	2	5
8	18	45	86	85	75	75	2	7
9	18	35	90	88	81	83	1	6
1o	18	40	82	85	70	72	1	7
11	18	40	83	85	67	70	2	6
12	18	45	85	86	65	65	1	7

Range of motion: the range of motion of the fifth metacarpophalangeal joint; *TIS* the injuried side, *TCS* the contralateral side

**Table 3:** Statistical analysis results of clinical and functional outcomes

n	Follow up time (months)	surgery duration (min)	Preoperative deformity		Postoperative deformity		Range of motion (°)		Grip strength (LB)		QDASH score	Return To Work (weeks)
			angulation (°)	length (mm)	angulation (°)	length (mm)	TIS	TCS	TIS	TCS		
12	16.8 ± 1.5	44.8 ± 5.4	40.0 ± 3.7	51.5 ± 2.1	17.6 ± 1.7	60.0 ± 1.8	84.3 ± 3.6	86.5 ± 2.0	74.8 ± 6.1	78.6 ± 8.3	2.0 ± 1.0	6.0 ± 0.7

Length: the length of the fifth metacarpal; Range of motion: the range of motion of the fifth metacarpophalangeal joint; *TIS* the injuried side, *TCS* the contralateral side

## Discussion

The surgical indications for the fifth metacarpal neck fracture have always been controversial [11–13]. For the fifth metacarpal neck fractures with angulation more than 30°, most surgeons agree that surgical treatment is more effective than conservative treatment [14, 15]. Whether the Kirschner wire fixation alone can provide sufficient fixation strength still remains controversial [16]. However, early function exercise needs to be based on a reliable internal fixation. With the traditional plate internal fixation, at least 2 screws are required for the distal part of the fracture to ensure the fixation strength. However, the purpose of this study was not to determine the threshold of fracture deformity for surgical treatment, but to analyze the curative effects of the modified internal fixation method for the treatment of fifth metacarpal neck fracture with angulation more than 30°. In this study, the mean angulation returned from 40.0° preoperatively to 17.6° postoperatively, while the length of the fifth metacarpal bone returned from 51.5 mm preoperatively to 60.0 mm postoperatively. Good recovery of the angulation and the length of the fifth metacarpal ensured that the range of motion of the fifth metacarpophalangeal joint and grip strength was not significantly different between the injured and contralateral sides, which also laterally confirmed the value of surgical treatment when the angulation is greater than 30°.

After the skin was cut and the fracture was exposed, surgeons needed to remove the bone hyperplasia and expose the fracture line clearly. Anatomical reduction could often be achieved by manipulative reduction by pulling the little finger and flexing the metacarpophalangeal joint. Once the surgeons were familiar with these procedures, it was not particularly difficult to realize the reduction of the fracture. The real challenge was to maintain long-term and stable fracture reduction, so reliable fixation with sufficient strength was very important. In this study, the cross-fixation of two Kirschner wires could not only strengthen the fixation strength of the fracture, but also temporarily maintain the fracture reduction, so that the placement of the plate got simple. Therefore, it was of great value that the Kirschner wires were satisfactorily implanted from the head of the metacarpal. In this study, two 1.0-mm Kirschner wires were used. Too thin Kirschner wires might not supply sufficient fixation strength, and too thick Kirschner wires might narrow the placement of the distal screw of the steel plate.

When the Kirschner wire was inserted, maintaining the flexion position of the metacarpophalangeal joint was conducive to the stability of fracture reduction. After the Kirschner wire broke through the contralateral bone, the tail external to the head of the metacarpal must be kept as short as possible to reduce the influences on joint activity as far as possible. After Kirschner wire insertion, C-arm fluoroscopy should be performed to confirm that the fracture reduction had been done before placing the plate. Proper plate plasticity was beneficial to the bonding of plate and metacarpal bone. A small portion of the capsule could be opened to allow the placement of the distal screw. If the joint capsule was opened partially, it should be sutured and repaired. The Kirschner wires were embedded under the skin to minimize the possibility of wound infection. After the fracture got healed, the internal fixation was removed under local anesthesia. Even though all patients underwent early function exercise under the guidance of the physiotherapists, we discovered some degree of tendon adhesion in all cases during the second surgery. Therefore, we performed arthrolysis and tendonolysis simultaneously during the second surgery. When the internal fixation was removed, a satisfactory range of motion could often be achieved by continuing with function exercise.

Compared with other surgical methods [17–20], this modified internal fixation method for the treatment of fifth metacarpal neck fracture has the following advantages: open reduction can ensure the quality of fracture reduction; the fixation strength is reliable, and can ensure the safety of early functional exercise after operation; all internal fixations are embedded subcutaneously to avoid the possibility of pin track infection; it has little effects on the metacarpophalangeal joint capsule and does not damage other tissues near the joint; for the fifth metacarpal neck fracture of which the distal bone is too small for two screws, the fixation strength can also be ensured; every patient is able to move his or her hand intraoperatively, and the surgeon can observe whether the joint movement is limited and adjust the operation process accordingly; the arthrolysis and tendonolysis can be performed simultaneously with the removal of the internal fixation. The disadvantages of this method mainly include the need of a second surgery and part of the joint capsule may be opened during the operation. Compared with the method of intramedullary anterograde Kirschner wires, this method results in skin scar that affects appearance [21].

In conclusion, this method is one of the alternative treatments for the fifth metacarpal neck fracture with good curative effects. In the treatment process, attention should be attached to the following problems: the indications of operation should be held strictly, and open reduction can be considered if the closed reduction fails; the Kirschner wires should be cross-fixed after satisfactory fracture reduction; the tail of the Kirschner wire should be kept as short as possible; when placing the plate, the surgeons should act gently to avoid the loss of fracture reduction; minimizing the opening range of the joint capsule; regular function exercise must be performed throughout the whole treatment process.

The small sample size and the lack of comparison with other surgical methods are the main deficiencies of this study. In the following work, the sample size will be further increased and comparative studies will be conducted as well.

## Data Availability

All data analyzed during this study are included in this published article.
